# Advanced technologies in InGaN micro-LED fabrication to mitigate the sidewall effect

**DOI:** 10.1038/s41377-025-01751-y

**Published:** 2025-01-26

**Authors:** Zhiyuan Liu, Haicheng Cao, Xiao Tang, Tingang Liu, Yi Lu, Zixian Jiang, Na Xiao, Xiaohang Li

**Affiliations:** Advanced Semiconductor Laboratory, Electrical and Computer Engineering Program, CEMSE Division, King Abdullah University of Science and Technology (KAUST), Thuwal, 23955-6900 Kingdom of Saudi Arabia

**Keywords:** Inorganic LEDs, Electronics, photonics and device physics

## Abstract

The size of InGaN micro-LEDs is continuously decreasing to meet the demands of various emerging applications, especially in tiny micro-displays such as AR/VR. However, the conventional pixel definition based on plasma etching significantly damages the mesa sidewalls, leading to a severe reduction in efficiency as the micro-LED size decreases. This seriously impedes the development and application of micro-LEDs. In this work, we comprehensively explain the origin of micro-LED sidewall effects and corresponding physical models. Subsequently, we systematically review recent progress in micro-LED fabrication aiming at suppressing sidewall effects. Furthermore, we discuss advancements in micro-LED fabrication with “damage-free” techniques, which hold the potential to fundamentally address the issue of plasma damage in the micro-LED process. We believe this review will deepen the understanding of micro-LED sidewall effects and provide a better insight into the latest associated fabrication technologies for high-efficient InGaN micro-LEDs.

## Abstract (web summary)

This work reviews the origins and mitigation strategies for sidewall effects in InGaN-based micro-LEDs.

## Graphical abstract

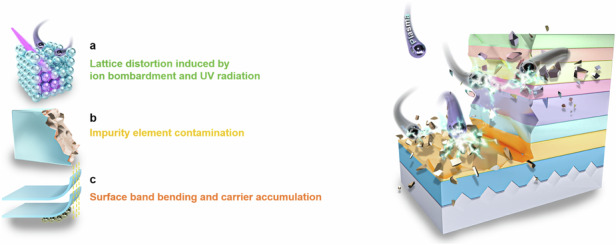

## Introduction

Recently, InGaN-based micro-LEDs have garnered considerable attention and advancement due to their remarkable characteristics, including high contrast, intense brightness, wide color gamut, and extended lifespan, making them promising contenders for next-generation display technology^1–5^. The reduction in the micro-LED size achieves a higher display resolution and brings many positive impacts on the device performance.

Due to the reduction in the mesa size, the current spreading in micro-LEDs becomes more uniform, effectively suppressing current crowding. This lowers the device series resistance thus smaller-size LEDs exhibit higher current density at the same operation voltage^6^. Furthermore, the reduced current crowding results in a more uniform heat generation, enhancing the overall heat dissipation capability. The specific thermal resistance decreases with the device size reduction. Therefore, smaller-sized LEDs exhibit lower junction temperatures at the same injection level, effectively suppressing self-heating effects^7^. Due to the reduced series resistance and self-heating effects, micro-LEDs can sustain higher current densities, which results in a higher carrier concentration in the quantum wells. Consequently, the effective carrier lifetime decreases, effectively enhancing the modulation bandwidth in visible light communication^8,9^. Furthermore, Ley et al. reported that smaller mesa size increased sidewall reflections and light directionality, contributing to less total internal reflection losses in the substrate^10^. A higher light extraction efficiency (LEE) thus could be obtained. The improvement of LEE by scaling mesa sizes has been observed in other reports as well^11–16^. Besides, due to the partial removal of the active region, strain is partially relaxed at the edge of the mesa^17–21^. The strain relaxation helps eliminate the quantum confined stark effect (QCSE) and enhances the radiative recombination rate. The impact of strain relaxation on the device performance becomes more pronounced as the mesa size decreases^22^.

**Fig. 1:**
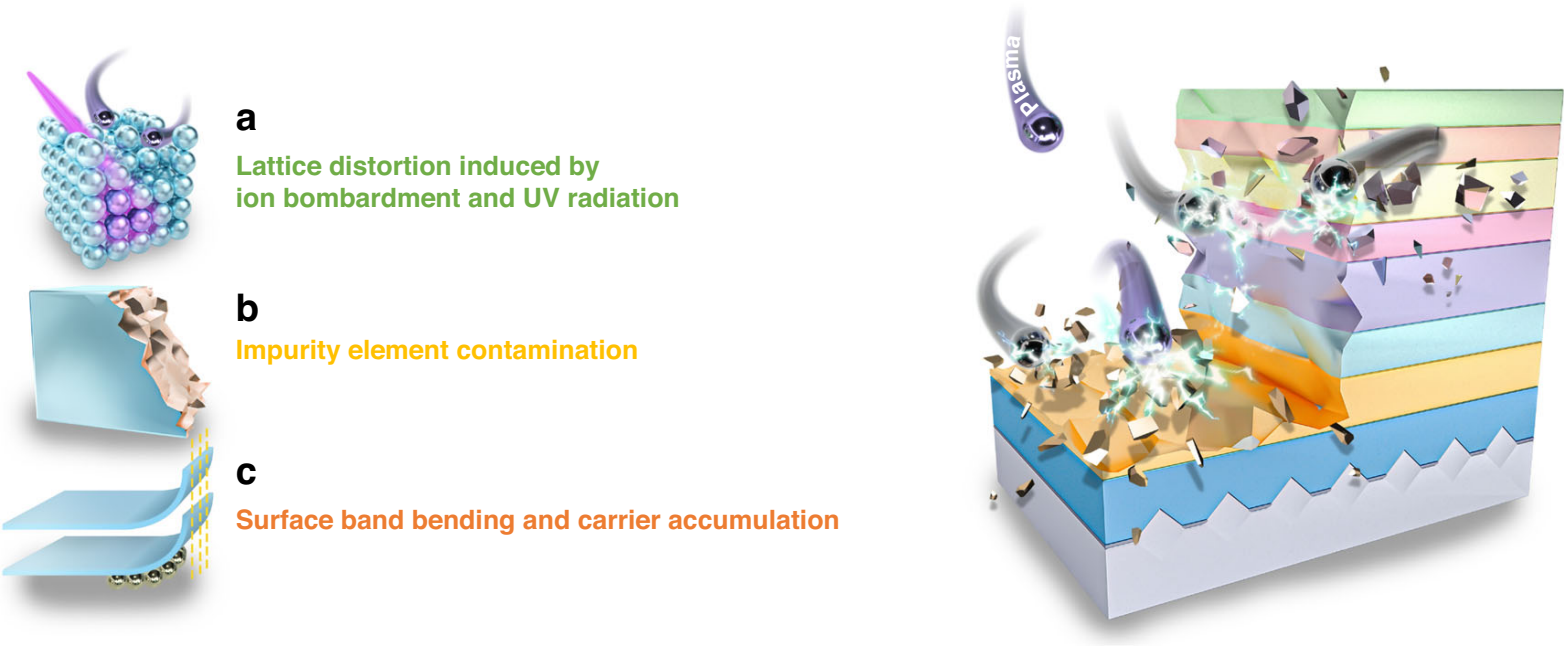
Schematic diagram of micro-LED sidewall damage. **a** Lattice distortion, **b** impurity element contamination, and **c** surface band bending and carrier accumulation

Nevertheless, the adverse effects of reducing the micro-LED size cannot be ignored. It is referred to as the “micro-LED sidewall effect,” closely tied to the fabrication processes of micro-LEDs. The conventional micro-LED fabrication process is based on plasma etching. Inductively coupled plasma (ICP) and reactive ion etching (RIE) are employed to define the micro-LED mesa and remove the excess active region. However, during the process, the plasma bombardment and UV radiation on the mesa sidewall can lead to significant defect formation, such as lattice distortion and impurity contamination, known as sidewall plasma damage, as shown in Fig. 1a, b^6,23^. These defects serve as current leakage paths and Shockley–Read–Hall (SRH) non-radiative recombination centers, severely limiting the device efficiency^24–27^. Furthermore, the sidewall surface states exacerbate surface band bending and accumulate carriers (Fig. 1 (c)), resulting in more severe surface recombination. The micro-LED sidewall effect becomes more pronounced with decreased mesa sizes, contributing to a size-dependence leakage current density and external quantum efficiency (EQE)^28–30^. Meanwhile, some literature reported that the current density at the EQE maximum was higher in smaller-sized micro-LEDs^31^, which will be detrimental to the micro-LED efficiency when devices are driven at low current densities. In this work, we explore the origins of micro-LED sidewall effects and provide a detailed review of recent innovations in micro-LED fabrication technologies to mitigate sidewall effects.

From an economic and commercialization standpoint, the production cost of micro-LEDs remains high due to factors such as chip manufacturing, mass transfer, packaging, and testing. Additionally, challenges persist in areas like yield control, uniformity, driver circuit architecture, and full-color display. However, we believe that continued research in micro-LED technology will gradually address these technical hurdles. In this review, we will not explore these aspects in detail.

## Micro-LED sidewall plasma damage

### Experimental observation of plasma damage on the surface

**Fig. 2:**
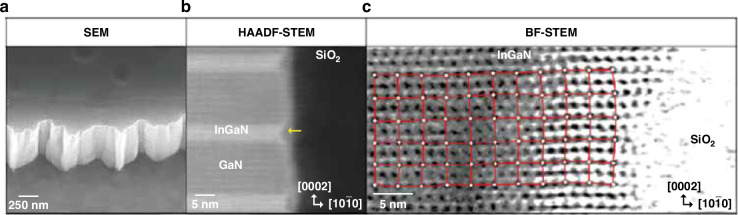
Lattice distortion near the micro-LED mesa sidewall. **a** SEM image of the m-plane sidewall, **b** HAADF-STEM image at the MQW/SiO_2_ interface, **c** BF-STEM image of lattice arrangement at the MQW/SiO_2_ interface. Reproduced with permission from ref. ^32^ © 2023, John Wiley and Sons

Plasma etching will induce severe lattice disorder near the mesa sidewall. Park et al. found atomic arrangement distortions near the sidewalls, with a total width of approximately ten lattice constants, as shown in Fig. 2a–c^32^. Meanwhile, lattice damage became increasingly evident closer to the sidewall surface. Yang et al. revealed lattice disorder regions of approximately 2 nm on the sidewall surface using high-resolution transmission electron microscopy (HRTEM)^33^. An amorphous lattice was found near the sidewall. Oxygen and silicon contaminations were also observed through the energy-dispersive X-ray (EDX) spectrum. The lattice distortions and surface contamination contributed to defect level formation, leading to SRH non-radiative recombination. Besides, Son et al. reported that the crystalline structure exhibited blurriness at the mesa sidewall, which was attributed to residual O atoms and lattice spacing variation^34^. The above results indicate that the plasma etching could damage the lattice structure, and the damage is most severe in the regions close to the sidewall surface. Therefore, surface treatment and passivation of the micro-LED sidewall are crucial for enhancing device performance.

**Fig. 3:**
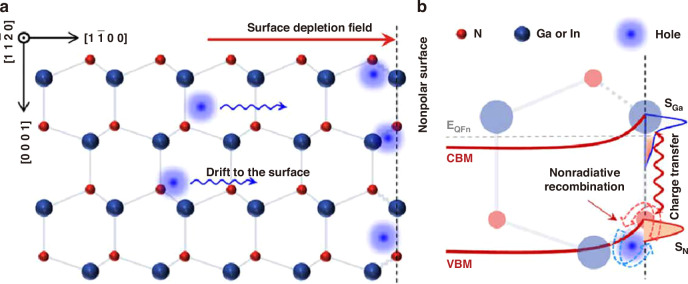
The surface recombination model. Schematic of (**a**) lattice structure and (**b**) band diagram near the mesa sidewall. Reproduced with permission from ref. ^35^ © 2020, John Wiley and Sons

Surface band bending also has a critical impact on micro-LED performance. Even without the plasma damage to the sidewall, surface band bending arises from intrinsic surface states at the lattice termination. These surface states mainly originate from dangling bonds^35,36^. Figure 3a, b illustrate a schematic diagram of the surface recombination model from Jiang et al. ^35^. Attributed to the energy disparity between the bulk Fermi level and surface states (Fermi-pinning), electrons diffuse to the surface and occupy the Ga dangling bonds. It leads to surface depletion and upward band bending. From the theoretical calculation, the band bending energy (*V*_bb_) is 0.6 eV, and the depletion region depth is 40 nm. Due to the existence of the surface depletion field, holes drift and accumulate at the surface. The accumulated holes result in the significant nonradiative recombination with electrons at N-deprived states. Furthermore, other defects on the sidewall surface, including lattice disorder, contamination, native oxide formation, etc., may increase the surface state density, aggravating the surface band bending and hole accumulation^32,37–39^. Besides, Li et al. found that after ICP etching, a microscopic strain field appeared at the sidewall, which influenced and modulated the surface band diagram^40^.

Furthermore, plasma-induced damage to the sidewall is penetrative. Intense ion bombardment can penetrate the material interior, and the penetration depth depends on the experiment condition, such as etching power and plasma sources^41^. Cao et al. demonstrated a damage depth of around 40 nm under 750 W source power and 150 W RF chuck power on the p-GaN sample. The damage depth was evaluated by the NaOH wet etching, where the etch rate was faster in disordered GaN than in the crystalline^42^. Yamada et al. reported a damage depth of less than 50 nm under an etching bias power of 30 W, which was determined by photo-electrochemical techniques^43^. More recently, Yamada et al. reported a 60 nm plasma damage depth was found on the n-GaN under the etching bias power 60 W, characterized by the capacitance-voltage measurement^44^. Moreover, the penetration of plasma-induced damage may also be associated with defect diffusion. Nakano et al. showed a carrier concentration reduction at 50 to 150 nm depth from the n-GaN surface, which was likely attributed to the diffusion of gallium vacancies (*V*_Ga_) into the bulk after suffering the surface ion bombardment^45^. Haberer et al. also found defect annihilation and defect diffusion phenomena by observing the PL intensity variation of shallow and deep quantum wells with surface GaN etching^46^. Besides, Minami et al. proposed that the UV photon irradiation from the plasma was more penetrative than the physics damage by ions. The former could have a penetration depth of more than 60 nm and generate defects, while the latter was within 2 nm^47^. The above results demonstrate that plasma etching will create a damaged region within a certain depth away from the material surface. Merely passivating the material surface is insufficient to eliminate all effects of etching damage. Therefore, removing the damaged region is crucial when fabricating micro-LEDs.

**Fig. 4:**
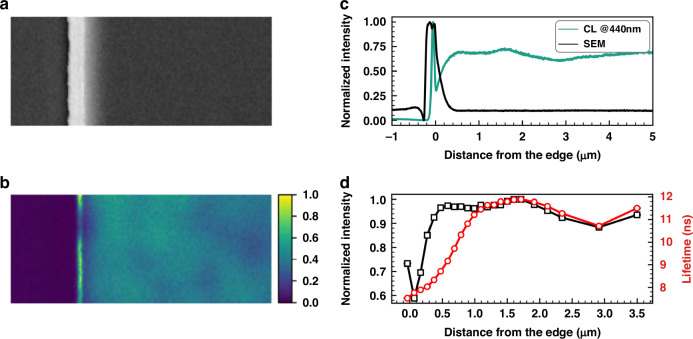
CL intensity and lifetime distribution on the micro-LED mesa. **a** SEM image at the edge of a micro-LED mesa (1 μm scale bar), **b** corresponding CL intensity distribution, **c** intensity of secondary electron (SE) and CL from the mesa edge to the center, **d** CL intensity and lifetime from the mesa edge to the center. Reproduced with permission from ref. ^48^ © 2022, American Chemical Society

**Fig. 5:**
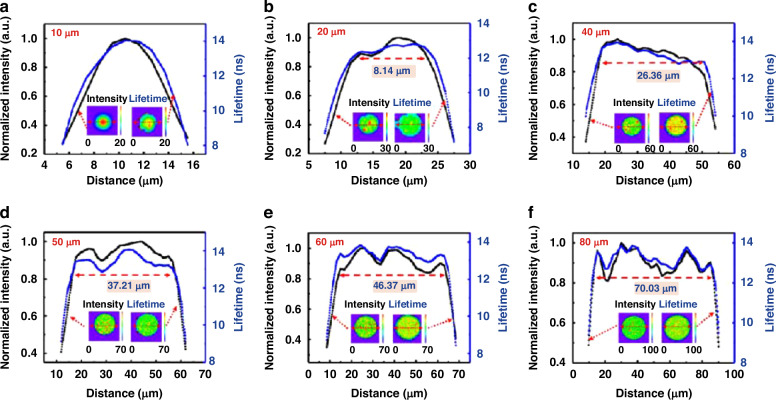
Fluorescence intensity and carrier lifetime distribution on the micro-LED mesa. Micro-LEDs with sizes of (**a**) 10 *μ*m, (**b**) 20 *μ*m, (**c**) 40 *μ*m, (**d**) 50 *μ*m, (**e**) 60 *μ*m, and (**f**) 80 *μ*m. Reproduced with permission from ref. ^49^ © 2023, Optical Society of America

The effect of plasma damage on micro-LEDs can be reflected by the emission intensity distribution on the mesa. Common techniques for such characterization include photoluminescence (PL), electroluminescence (EL), cathodoluminescence (CL), fluorescence, laser confocal scanning microscope (LCSM), etc^33^. Finot et al. demonstrated the CL intensity and CL lifetime distribution on the mesa, as shown in Fig. 4a–d^48^. It was initially determined through simulation that the impact of strain relaxation on carrier lifetime should exist within 200 nm from the mesa edge. However, from the CL lifetime profile, the carrier lifetime was lower than the center region within a range of approximately 1 μm from the edge. This exceeded the influence range of strain relaxation. Therefore, non-radiative recombination through surface defects played a significant role here. Meanwhile, around 450 nm to 1 μm from the edge, the CL intensity remained constant while the carrier lifetime increased. It was explained that the light extraction efficiency was enhanced at the mesa edge. Wang et al. utilized a fluorescence technique to observe the mesa area affected by sidewall defects. As depicted in Fig. 5a–f, fluorescence intensity and carrier lifetime rapidly decreased at the mesa edge. The affected width exceeded 5 μm on one side. Therefore, as the size of the micro-LED was reduced to 10 μm, nearly the entire mesa region was influenced by sidewall defects^49^. Yang et al. employed PL measurement to character emission intensity, wavelength, FWHM, and EQE from the mesa edge to the center. The region impacted by plasma damage was determined to have a width of less than 800 nm^50^. Besides, numerous studies have also confirmed the emission degradation near the mesa edge. The degradation reached a nominal range of several hundred nanometers to a few micrometers^16,51–55^. Dramatic surface defects and carrier diffusion to the sidewall were significant factors for such a severe impact.

In summary, plasma etching-induced damage to the micro-LED sidewall is an extremely complex process, encompassing lattice distortion, impurity contamination, surface band bending, and the penetrative nature of the damage. The dominance of different factors and their impact on device performance relies on process conditions. However, it can be affirmed that sidewall effects become more pronounced in smaller-sized micro-LEDs (e.g., below 10 μm) due to the larger ratio of sidewall perimeter to mesa area. The micro-LED efficiency will be significantly suppressed without proper treatment and passivation after plasma etching.

### Model and formulate the sidewall damage

The working of micro-LEDs follows the laws of device physics, such as carrier transport and carrier recombination equations. Meanwhile, establishing equivalent or simplified models and formulating the impact of plasma damage on micro-LEDs is crucial for better understanding the sidewall effect. Recently, the concept of a “dead zone” has been proposed for modeling the sidewall damage of micro-LEDs. The dead zone is an equivalent model of the region affected by plasma damage. As an equivalent model, the defect density, surface recombination rate, carrier diffusion, etc., should be considered when determining the dead zone width. Different fabrication processes will also fundamentally influence the modeling.

**Fig. 6:**
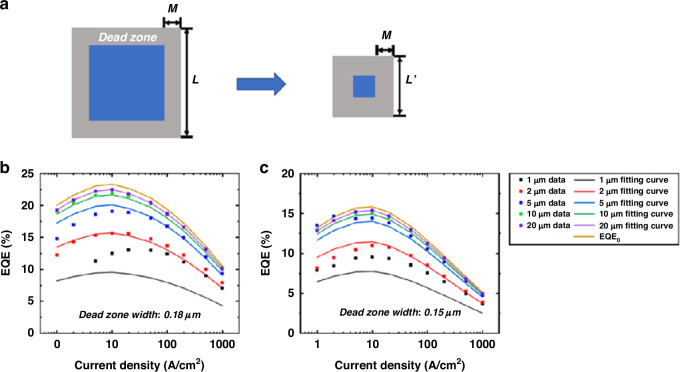
Micro-LED “dead zone” model. **a** Schematic diagram of micro-LED “dead zone,” measured EQE of (**b**) blue micro-LEDs and (**c**) green micro-LEDs and corresponding fitting curves according to the “dead zone” model. Reproduced with permission from ref. ^56^ © 2022, AIP Publishing

Yu et al. proposed Eq. 1 to calculate the micro-LED *EQE* based on the built “dead zone” model shown in Fig. 6a, where *L* was the mesa size, *M* was the dead zone width, *EQE*_*0*_ was the external quantum efficiency without considering any dead zone^56^.

1 $$\,{EQE}={{EQE}}_{0}\times \frac{{(L-2M)}^{2}}{{(L)}^{2}}$$

In this model, the current was uniformly injected into both the “dead zone” and the un-damaged region, but the “dead zone” did not contribute to any emission. This means all carrier recombination in the “dead zone” is non-radiative. Therefore, as the micro-LED size decreased, the *EQE* would decrease proportionally with the increase in the area ratio of the “dead zone” to the un-damaged region. Through the above model, The efficiency of 2 to 20 μm blue and green micro-LEDs was accurately assessed, and well-fitted with the experiment dates, as shown in Fig. 6b, c. The calculated “dead zone” width was 180 nm for blue micro-LEDs and 150 nm for green micro-LEDs. The width of the dead zone can also be used to evaluate the size limitation of micro-LEDs fabricated based on the plasma etching process. However, in their study, the current density at the *EQE* maximum did not obviously increase as the micro-LED size decreased, although the efficiency was continuously reduced. In other words, the above model may not be applicable to experimental data depicting the *EQE* peak shifting with sizes. The introduction of the modified IQE-ABC model may better describe this characteristic.

2 $$\,{EQE}={IQE}\times {LEE}=\frac{{{Bn}}^{2}}{{An}+{{Bn}}^{2}+{{Cn}}^{3}}\times {LEE}$$

3 $${n}_{{IQE}-{peak}}=\sqrt{\frac{A}{C}}$$

4 $${{IQE}}_{{peak}}=\frac{\frac{B}{\sqrt{{AC}}}}{\frac{B}{\sqrt{{AC}}}+2}$$

5 $$\,A={A}_{0}+\frac{{A}_{s}\lambda l}{S}$$

The modified IQE-ABC model for micro-LEDs has been described recently in several reports, as shown in Eq. 2^7,15,32^. *EQE* is the external quantum efficiency. *IQE* is the internal quantum efficiency. *LEE* is the light extraction efficiency. *A*, *B*, and *C* are rate constants of SRH non-radiative recombination rate, radiative recombination rate, and Auger non-radiative recombination rate, respectively. *n* is the carrier density. If temporally considering the *LEE* as a constant, by differentiating the *EQE* (*IQE*) equation, one will get the carrier density *n*_IQE-peak_ when the *EQE* (*IQE*) reaches the peak value, as shown in Eq. 3. It implies that with a larger *A* coefficient, the *EQE* (*IQE*) peak will correspond to a higher carrier concentration, i.e., higher current density. This explains the origin of the higher current density at the *EQE* maximum when micro-LEDs suffer more severe surface recombination. Besides, the larger *A* coefficient will also lead to the suppression of peak efficiency, as shown in Eq. 4. Due to the sidewall damage of micro-LEDs, the SRH non-radiative recombination rate constant *A* needs to be adjusted. As described in Eq. 5, the modified coefficient includes two terms. *A*_0_ is an SRH non-radiative recombination rate constant in the bulk region without being affected by the plasma damage. Another part is to consider the impact of surface recombination. In the equation, *A*_s_ is the surface non-radiative recombination rate constant, *λ* is the carrier diffusion length, *l* is the peripheral length, and *S* is the mesa area of micro-LEDs. With more plasma-induced surface damage, *A*_s_ value will increase, contributing to more non-radiative recombination at the sidewall. Simultaneously, the SRH non-radiative recombination rate will become more prominent as the device size decreases, which has been well demonstrated by Oliver and Lee et al.^57,58^. This phenomenon is manifested in the ratio of perimeter *l* to area *S*. In addition, the carrier diffusion length *λ* should not be overlooked. More carriers reaching the defect-rich sidewall will lead to more severe non-radiative recombination. Therefore, the carrier diffusion length should also be considered in the equation. After accounting for the additional contribution from surface recombination, the total SRH non-radiative recombination rate constant *A* increases, leading to a lower *EQE* and higher current density at the *EQE* maximum.

**Fig. 7:**
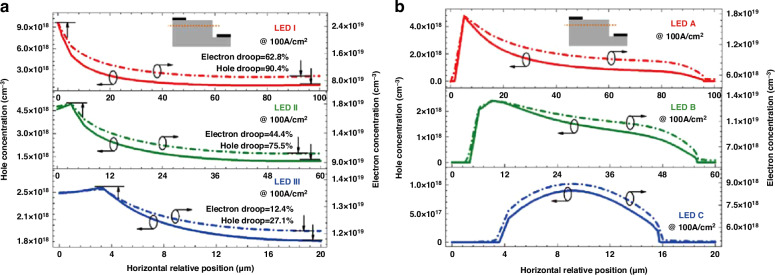
Modeling and simulation of the micro-LED sidewall effect. Calculated carrier concentration distribution in the quantum well (the one adjacent to the p-side) of (**a**) LED I (100 µm), LED II (60 µm), and LED III (20 µm) without “dead zone” and (**b**) LED A (100 µm), LED B (60 µm), and LED C (20 µm) with “dead zone.” Reproduced with permission from ref. ^66^ © 2019, Optical Society of America

Numerical simulation is a valuable tool for theoretical calculations to predict device performance, analyze device physics, and optimize the device structure. Recently, numerous device simulation studies focusing on the micro-LED and its sidewall effects have been reported^7,27,59–65^. Impressively, Kou et al. employed the APSYS simulation software to study the effects of surface recombination on blue micro-LEDs^66^. In this work, a “dead zone” with a width of 4 μm was modeled. The electron trap level was set at “*E*_c_ − 0.24 eV” with a capture cross-section of 3.4 × 10^−17^ cm^2^ and a density of 10^13^ cm^−3^. The hole trap level was defined at “*E*_v_ + 0.46 eV” with a capture cross-section of 2.1 × 10^−15^ cm^2^ and a density of 1.6 × 10^13^cm^−3^. Through the above model, micro-LED characteristics such as the I-V, EQE curves, and carrier distribution were simulated. It was observed that the sidewall defects formed current leakage channels. At low forward voltages, micro-LEDs with dead zones showed a higher leakage current than those without damage zones. Simultaneously, due to the leakage path, when comparing micro-LEDs of the same size with and without plasma damage, the damaged micro-LED showed elevated current density at an identical operating voltage. Besides, it was observed that sidewall defects could capture both electrons and holes, facilitating SRH recombination. This simultaneously led to a reduced carrier concentration within the bulk MQWs of damaged micro-LEDs, as evidenced by the comparison between Fig. 7a, b. The trapping effect was more significant for holes than for electrons, attributed to their larger capture cross-section, higher trap density, and lower mobility. Therefore, not only are sidewall treatment and passivation crucial in micro-LEDs, but enhancing carrier injection capability (especially for holes) should not be ignored.

In summary, creating proper models and formulas on sidewall damage is crucial for a deeper understanding of micro-LED sidewall effects and optimizing device performance. Currently, people have understood some typical behaviors of micro-LEDs, such as I-V characteristics and efficiency curves. The precision of the model description can still be further improved in the future to encompass the difference in results under different experimental conditions. Besides, simulation methods such as finite element calculation can predict micro-LED performance precisely. It is also straightforward to observe trends. However, the accuracy of structure modeling and parameter settings is crucial for the results. This requires more research by referring to experimental data.

## Micro-LED process to eliminate the sidewall effect

As mentioned above, the sidewall effects of micro-LEDs cause significant suppression of device performance. It originates from lattice distortion, contamination, surface band bending, carrier diffusion, etc. Targeted suppression of these issues can be achieved through designing and optimizing the fabrication process. According to existing reports, we categorize optimization methods into three types: (1) Remove the surface damaged layer; (2) Passivate the sidewall surface; (3) Control the carrier transport path.

### Remove the surface damaged layer

Plasma induces severe lattice distortion on the sidewall surface, leading to numerous defects for non-radiative recombination and deteriorating surface band bending. This region can be removed through chemical treatments. Since chemical reagents can usually etch to a certain depth, they are effective in removing the penetrative damage (inside the sidewall) caused by UV irradiation and ion bombardment. TMAH and KOH are hydroxyl groups (−OH) chemicals commonly used in III-nitride-based device processes^67,68^. They are difficult to react with the GaN c-plane but can be utilized to etch the micro-LED sidewall (m-plane or a-plane). The etching rate depends on the density of planar atoms and the quantity of surface dangling bonds^69,70^. The crystal orientation of the micro-LED sidewalls may impact device performance. According to reports, the chemical treatment resulted in the m-plane becoming smooth and steep, while the a-plane ultimately developed a zigzag pattern through the formation of adjacent m-plane prism structures^15,71,72^. The prism structures may result in a larger sidewall surface area and more dangling bonds for the a-plane after TMAH and KOH treatment compared with the m-plane.

**Fig. 8:**
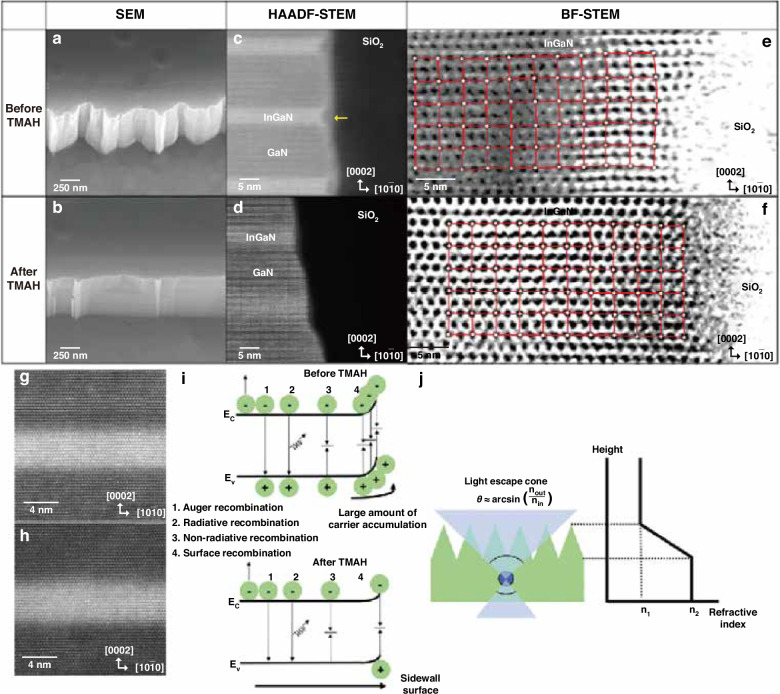
Micro-LED sidewall before and after TMAH treatment. SEM images of m-plane sidewalls (**a**) before and (**b**) after TMAH treatment, HAADF-STEM images at the MQW/SiO_2_ interface (**c**) before and (**d**) after TMAH treatment, BF-STEM images of lattice arrangement at the MQW/SiO_2_ interface (**e**) before and (**f**) after TMAH treatment, HAADF-STEM images of the quantum well and barrier far away from the sidewall (>300 nm) (**g**) before and (**h**) after TMAH treatment, (**i**) schematic diagram of carrier recombination near the mesa sidewall, (**j**) schematic diagram of light extraction enhancement enabled by the rough sidewall morphology. Reproduced with permission from ref. ^32^ © 2023, John Wiley and Sons

Park et al. characterized the m-plane sidewall morphology before and after TMAH treatment using SEM and TEM images, as shown in Fig. 8a–h^32^. It can be observed that, after the treatment, the sidewall became smooth and steep. The regions with lattice disorder were effectively removed. The treatment reduced the number of surface states, which acted as SRH non-radiative recombination centers. Simultaneously, suppressed surface states alleviate surface band bending and carrier accumulation, more effectively controlling the non-radiative recombination, as shown in Fig. 8i. Therefore, the IQE increased after the TMAH treatment. It was also demonstrated that the leakage paths at the sidewall were significantly reduced after the treatment^15^. However, as shown in Fig. 8j, the rough sidewall before the treatment may help the light extraction via sidewall emission. Therefore, sidewall smoothening may reduce the light extraction efficiency. As the micro-LED size becomes smaller, the impact of sidewall configurations on light extraction efficiency becomes more significant^73,74^. Therefore, it is crucial to consider and balance the IQE and LEE to enhance overall EQE. Besides, similar results about micro-LED sidewall treatment using TMAH and KOH have been reported in numerous literature sources^28,32,39,48,75–86^.

Sulfides, such as (NH_4_)_2_S, were reported for chemical treatment in III-Nitride and III-V devices^38,87^. The mechanism is to remove unstable native oxides to reduce surface states and form a monolayer of sulfides for surface passivation^88,89^. Choi et al. found that (NH_4_)_2_S treatment decreased leakage current and improved EL intensity in InGaN nanopillar LEDs by suppressing sidewall non-radiative recombination centers^90^. Leem et al. found similar results in InGaN LEDs with photonic crystals^91^. Yang et al. showed that the I-V characteristic could partially improve after the (NH_4_)_2_S treatment in the InGaN-based blue LED with micro-arrays. However, significant surface leakage still existed after the treatment. Therefore, (NH_4_)_2_S treatment was effective in passivating the surface state but it might be hard to remove the sidewall-damaged region completely^92^. Polyakov et al. observed a similar phenomenon to the one above. The simultaneous use of KOH and (NH_4_)_2_S treatment increased the PL intensity of plasma-damaged nano-pillars. However, excess leakage current still existed in the I-V characteristic and needed to improve through an extra thermal annealing process^93^. Maoult et al. revealed that the indium component in InGaN was preferentially etched in the (NH_4_)_2_S chemical. After the treatment, the surface adsorbed sulfur is in the range of 10^14^ at.cm^−2^ measured by the wavelength dispersive X-ray fluorescence technique. The sulfur was presumably bonded with gallium or indium by substitution of oxygen^94^. The utilization of sulfides for nitride surface treatment has been also shown in literature sources^95–99^. However, there are relatively few reports on the sulfide treatment on InGaN-based micro-LED devices, and more investigations are needed.

Besides, other chemicals such as H_3_PO_4_, NH_4_OH, HF, HCl, octadecylthiol, parylene, citric acid, etc., were reported for sidewall treatment to improve device performance by removing the lattice distortion, native oxide, and surface contaminants^38,39,94,100–106^. However, there are relatively few reports on the use of these chemical treatments on InGaN-based micro-LEDs. More in-depth investigations should be conducted to reveal their effect on device performance and underlying physical mechanisms.

### Passivate the sidewall surface

Regardless of plasma damage, the material surface always contains a significant number of dangling bonds. Passivating active dangling bonds helps reduce surface state density and prevent sidewall impurity contaminations from the external environment. This type of method mainly affects the defects on the sidewall surface and has weak treatment on the damage inside the sidewall (penetrative damage). Therefore, it can be combined with chemical solutions, etc. methods to remove a certain thickness of the damaged layer before proceeding with surface passivation.

**Fig. 9:**
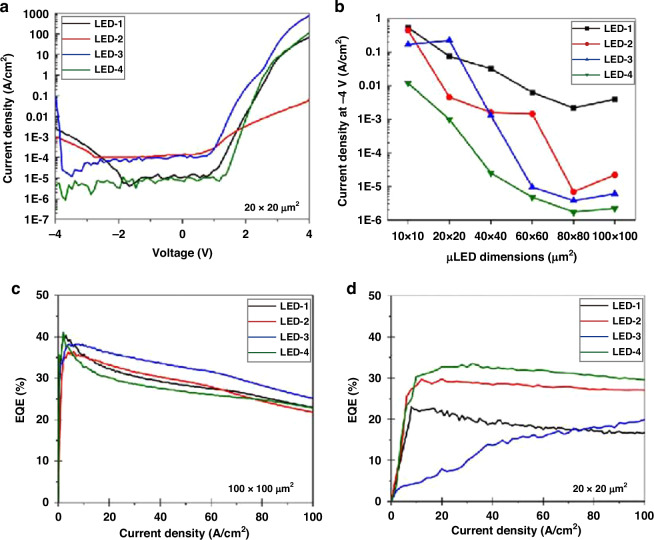
Micro-LED performance with ALD passivation. **a** I-V characteristics of 20 × 20 µm^2^ micro-LEDs with different sidewall passivation methods, **b** micro-LED leakage current at −4 V with different sizes and sidewall passivation methods, EQE for (**c**) 100 × 100 µm^2^ and (**d**) 20 × 20 µm^2^ devices with different sidewall passivation methods. Note: LED-1 without any sidewall passivation, LED-2 ALD passivation and aperture formation by ICP etching, LED-3 PECVD passivation and aperture formation by HF etching, LED-4 ALD passivation and aperture formation by HF etching. Reproduced with permission from ref. ^115^ © 2018, Optical Society of America

Depositing dielectric materials on the micro-LED sidewall is one of the most direct approaches to passivation. The SiO_2_ produced through plasma-enhanced chemical vapor deposition (PECVD) is extensively utilized for insulation and surface passivation in LEDs^107–113^. As a potential alternative method, the ALD technique can achieve high-quality and uniform film, precise atomic-level thickness control, and excellent step coverage^114^. Recently, Wong et al. utilized the ALD method for micro-LED sidewall passivation^115^. Figure 9a–d compared the leakage current and EQE of devices passivated with PECVD-SiO_2_ and ALD-Al_2_O_3_. It was found that compared with PECVD-SiO_2_, ALD-Al_2_O_3_ yielded better passivation effects, reducing leakage current and improving EQE for micro-LEDs. The improvement from the ALD deposition method was related to superior dielectric material quality compared to PECVD-SiO_2_. Besides, during the PECVD-SiO_2_ process, the presence of hydrogen radicals in the precursor reduced the transparency of ITO. The use of metalorganic precursors in ALD could avoid this issue. Lee et al. utilized the IQE-ABC model to extract and compare the SRH coefficients between micro-LEDs with or without the passivation of ALD-Al_2_O_3_^58^. The surface recombination velocity and SRH coefficient were significantly reduced in ALD-passivated devices. Furthermore, Lai et al. found that the EL intensity in the ALD-passivated device had a lower drop from the mesa center to the edge. This indicated that the surface recombination was suppressed. More carriers thus could be injected into the bulk region instead of being trapped by the surface defects. The device with ALD passivation also improved the EQE and VLC performance^116^. Moreover, Lee and Kang et al. mentioned that the use of plasma in the PECVD may bring additional sidewall damage during passivation^58,117^. This effect may be mitigated or avoided during the ALD process. Besides, Huang et al. demonstrated a longer device lifetime in ALD-SiO_2_ passivated AlInGaP micro-red LED compared with the PECVD-SiO_2_ one^118^. However, the effectiveness of ALD passivation in prolonging the device lifetime of InGaN-based micro-LEDs still needs to be validated. Due to the excellent performance of ALD in passivation, it is becoming a mainstream method for micro-LED passivation, widely applied in various research in the community.

The above results proved the role of ALD in micro-LED sidewall passivation. However, the impact of different materials prepared by the ALD on the micro-LED performance needs to be studied. Son et al. investigated the effects of ALD-SiO_2_, Al_2_O_3_, and Si_3_N_4_ on the sidewall passivation of micro-LEDs^34^. It was found that SiO_2_ owned superior passivation performance compared to Al_2_O_3_ and Si_3_N_4_. Passivation using ALD-SiO_2_ resulted in a lower leakage current, lower device resistance, and higher efficiency. Through XPS analysis, It was concluded that the Ga–O formation energy (545 kJ mol^−1^) was lower than the Si–O dissociation energy (799.6 kJ mol^−1^). Therefore, O atoms could diffuse from the air into the sidewall interface during the subsequent annealing process, passing through the SiO_2_ without any chemical reaction with Si or O species. The arriving O atoms helped passivate Ga dangling bonds and N vacancies, significantly reducing the leakage paths and surface non-radiative recombination. Chen et al. proposed that compared with widely used ALD-Al_2_O_3_ passivation, ALD-AlN showed a better passivation performance^119^. Such passivation contributed to a lower leakage current, higher operation current density under the same voltage, improved output power, and superior EQE. Ball and stick models were employed in their study to understand the above observations. It showed that the lattice mismatch of AlN/GaN was tinier than Al_2_O_3_/GaN, which resulted in a more homogeneous passivation with less Ga/In dangling bonds at the AlN passivated sidewall.

A multi-layer structure for passivation layers has also been proposed. Lee, Shen, and Yang et al. demonstrated ALD-Al_2_O_3_/PECVD-SiO_2_ double passivation layer structures in the micro-LED fabrication^24,114,120,121^. Ghods et al. proposed an Al_2_O_3_/ZnO passivation layer to modulate the electric field near the sidewall^122^. Cho et al. employed the triple dielectric layers, including thermal ALD-HfO_2_ or SiO_2_/plasma-enhanced ALD-SiO_2_/thermal ALD Al_2_O_3_, in their sub-micron size LEDs^123^. In fact, due to the relatively slow deposition rate of ALD compared with PECVD, the thickness of the ALD passivation layer is generally less than 50 nm. Therefore, utilizing PECVD to deposit an additional layer of thick dielectric material is advantageous for enhancing the electrical insulation of the device and reducing the risk of device failure^10^.

In addition, people have applied other methods for the passivation of micro-LEDs. Sheen et al. demonstrated that sol-gel-derived SiO_2_ exhibited superior passivation performance compared to plasma-enhanced ALD-SiO_2_ in nano-scale LEDs. The improvement was attributed to the adsorption process of SiO_2_ nanoparticles from the sol-gel onto the GaN surface. This minimized atomic interactions with the GaN surface and effectively passivated surface dangling bonds^124^. Huang et al. also demonstrated that as a low-cost fabrication process, sol-gel SiO_2_ sidewall passivation significantly improved device performance compared to the process without sidewall passivation and with PECVD-SiO_2_ passivation^125^. A combination of N_2_ plasma treatment and annealing processes was employed by Lee et al. to recover etch-induced damage in InGaN LEDs. The leakage current and EL intensity were significantly reduced and enhanced after the treatment, respectively^126^. Jin et al. introduced PECVD-N_2_ plasma treatment after the sidewall ICP-etching. Extra N atoms were incorporated into the surface, which reduced the number of Ga dangling bonds and improved micro-LED efficiency^127^. Liu et al. used the N ion to passivate the sidewall. The N ion effectively removed surface native oxide and occupied dangling bonds^128^. Xu et al. utilized N_2_O plasma treatment to suppress the sidewall non-radiative recombination^129^. The sidewall defects exposed to N_2_O were oxidized and passivated to form thermodynamically stable Ga-O species. Yang, Tian, Shin, and Polyakov et al. demonstrated the repair of the sidewall damage by a rapid thermal annealing method^31,92,93,130,131^.

### Control the carrier transport path

To reduce non-radiative recombination at the sidewall, one available approach is to confine more current and carrier density in the mesa center and away from the damaged area. This effect is achieved by controlling the current path, but it may not change the situation of sidewall defects. Therefore, it is better to use it in conjunction with the methods described in previous sections. Meanwhile, enhancing the overall hole injection capability through structure optimization will also contribute to better micro-LED performance^64,132^. Behrman et al. enhanced micro-LED efficiency by varying p-GaN contact geometries to modulate current spreading and surface recombination^133^. Chen et al. suppressed the hole spreading to the sidewall by thinning the p-GaN layer, significantly improving the micro-LED efficiency^134^. Huang et al. proposed the lateral oxide-confined structure to achieve current confinement. The p-GaN in the defined region was shallowly etched and refilled by the SiO_2_ to form the current aperture^135^. Similarly, Zhang et al. used Ta_2_O_5_ to refill the step after p ^+^ -GaN was selectively etched. The Ta_2_O_5_ has a large dielectric constant, contributing to a high hole barrier formation near the mesa edge to suppress the hole spreading and surface recombination^136^.

**Fig. 10:**
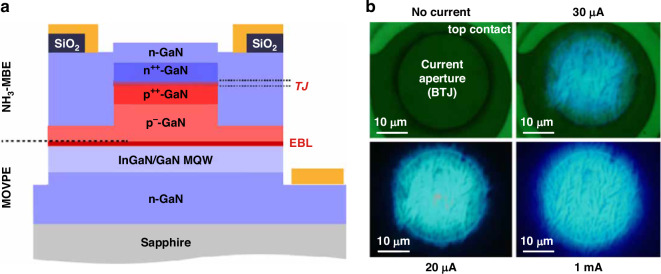
Current aperture formed by a buried tunnel junction. **a** Micro-LED structure with a buried tunnel junction, **b** EL images with different injection currents. Reproduced with permission from ref. ^137^ © 2015, AIP Publishing

Instead of utilizing dielectric oxide, a buried tunnel junction was demonstrated to form the current aperture in micro-LEDs by Malinverni et al., as shown in Fig. 10a, b^137^. The different characteristics of PN junctions with different doping levels were ingeniously exploited. For lightly doped PN junctions, they exhibited rectification character under reverse bias. In the case of heavily doped PN junctions, they formed a tunnel junction that conducted current under reverse bias. Based on the above mechanisms, a regrown n-GaN filled the etching step and covered the buried tunnel junction. At the mesa center, hole injection could be achieved by the highly doped tunneling junction n-GaN/n^++^-GaN/p^++^-GaN/ p^−^-GaN. At the meas edge, holes were blocked by a reversed bias p^−^-GaN/n-GaN junction, which helped suppress the surface recombination. Similarly, Hang et al. artificially formed a high-resistance ITO/p-GaN junction in GaN-based micro-LEDs to suppress the current spreading to the mesa sidewall^138^. The heavily doped p^+^-GaN layer near the sidewall (4 µm-width) was selectively removed and refilled by the ITO. The ITO in the mesa center formed an ohmic contact with p^+^-GaN while introducing a more resistive ITO/p-GaN junction near the mesa edge to prevent holes from reaching the sidewall. It was found that such a structure facilitated better performance for the 30 μm micro-LED than reference samples due to the suppressed surface recombination and better center hole injection after employing the current confinement structure. However, for the 100 μm device, the performance was worse than conventional micro-LEDs as a result of more serious current crowding and heat generation. Therefore, when using current confinement structures to suppress carrier movement toward the sidewalls and non-radiative recombination, people should consider the impact of current crowding on device performance.

**Fig. 11:**
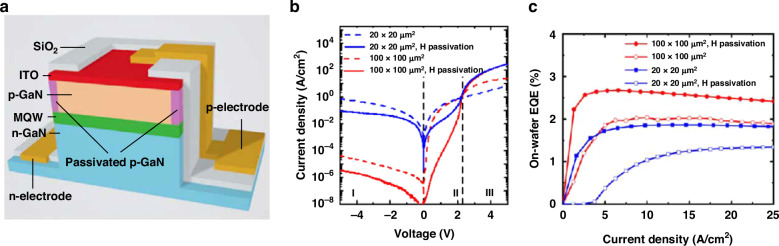
Micro-LED surface passivation by H_2_ plasma. **a** Micro-LED structure with H_2_ plasma sidewall passivation, **b** I–V characteristics of fabricated micro-LED arrays, **c** EQE curves of fabricated micro-LED arrays. Reproduced from ref. ^139^ © 2022, P. Kirilenko et al. under the terms of the Creative Commons Attribution 4.0 License

Pavel et al. demonstrated an H_2_ plasma passivation method in micro-LEDs, shown in Fig. 11a^139^. As a reverse process of Mg activation in p-GaN, H_2_ plasma treatment on the sidewall resulted in Mg-H complex formation, which made the sidewall p-GaN insulate to suppress surface conduction and non-radiative recombination. The ITO layer on the mesa top served as p-GaN contact and was also employed as a hard mask to block H_2_ plasma when the sidewall was under treatment. By H_2_ plasma passivation, the micro-LED leakage current was suppressed (Fig. 11b), and the efficiency was improved (Fig. 11c). Moreover, Yin et al. designed a metal-insulator-semiconductor structure by depositing an extra electrode on the sidewall. The voltage bias on the sidewall electrode modulated the sidewall electric field, which manipulated the surface carrier density and controlled surface non-radiative recombination^140^. Besides, although not directly related to the device fabrication, it is worth mentioning that the carrier localization in InGaN may positively impact suppressing surface recombination. Carriers accumulate in high indium clusters and keep away from defects to improve radiative efficiency. Smith and Liu et al. found that green micro-LED has a higher EQE than blue when the size became small enough. It was explained that the carrier localization was stronger in the higher indium InGaN, which helped reduce the surface recombination velocity^30,141^.

In summary, significant efforts have been devoted to suppressing the micro-LED sidewall effect and enhancing device efficiency. The proposed methods are artificially categorized into three types based on their main mechanisms. It should be noted that the three types of approaches have a certain degree of independence. Removing the sidewall damage layer, passivating the sidewall surface, and controlling the current path can contribute from different angles parallelly, improving micro-LED performance by mitigating sidewall effects. Therefore, many reports combined multiple treatment methods during the micro-LED process, such as chemical treatment + ALD passivation, selective etching current confinement + ALD passivation, chemical treatment + H_2_ plasma current confinement, etc.^28,136,139^. Additionally, the impacts of some methods are multifaceted. For instance, (NH_4_)_2_S may work both in removing unstable native oxides and forming a monolayer of sulfides for surface passivation. ALD-Ta_2_O_5_ serves as surface passivation and simultaneously contributes to a high hole barrier formation near the mesa edge to suppress the hole spreading and surface recombination, due to its large dielectric constant. Therefore, a multidimensional analysis is crucial when conducting “sidewall engineering”. We believe the micro-LED sidewall effect will be substantially suppressed with the enrichment of treatment methods and a deeper understanding of the physics mechanism. After eliminating the sidewall effect, micro-LED efficiency is highly anticipated due to numerous merits, such as better current spreading, suppressed self-heating effect, strain relaxation near the surface, and boosted light extraction.

## “Damage free” micro-LED process

The sidewall damage from the ICP-RIE process becomes more pronounced as the micro-LED size decreases. Although various treatments effectively improve micro-LED performance, it may struggle to completely avoid the effects of plasma-induced damage. Therefore, fabricating micro-LED pixels without conventional ICP-RIE process might be an alternative and promising solution. In this section, we will summarize and introduce these methods.

### Direct epitaxy methods

**Fig. 12:**
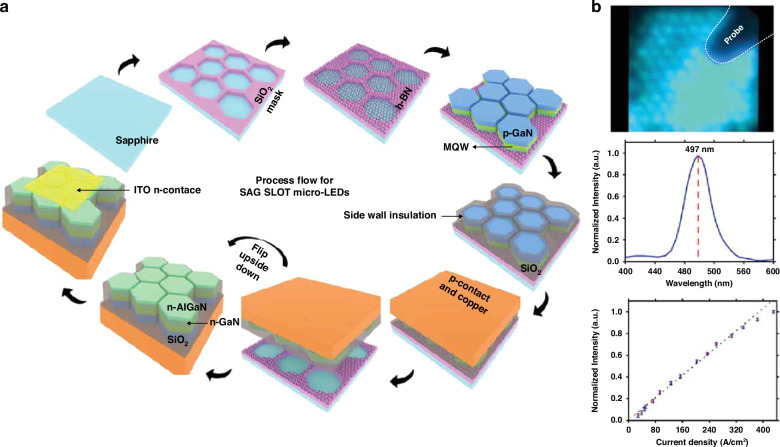
Micro-LEDs by selective area growth on the h-BN template. **a** Fabrication process flow, **b** near field image, EL spectrum, and normalized output power of 8 μm micro-LEDs. Reproduced with permission from ref. ^145^ © 2023, John Wiley and Sons

Selective area epitaxy or growth (SAE or SAG) is popular for various nanostructure formations in InGaN-based LEDs^142–144^. Recently, this approach has been utilized for micro-LED direct epitaxy. In the process, dielectric or metal masks are commonly employed to confine the epitaxy area. After epitaxial growth, micro-LED islands naturally isolate each other without the additional plasma etching to define pixels. Gujrati et al. demonstrated defect-free sidewall micro-LEDs by selective area growth on the h-BN template^145^. As shown in Fig. 12a, Patterned SiO_2_ served as the mask for micro-LED SAG. For the device fabrication, global p-contact and copper support were deposited on the p-GaN after the sidewall insulation. The h-BN deposited on the SiO_2_ mask formed vdW bonds instead of vertical chemical bonds with the epitaxy structure interface. This allowed the simple micro-LED lift-off and transfer. Utilizing the above process, down to 1.4 μm size micro-LED pixels have been grown, and micro-LEDs with sizes 8 to 100 μm have been successfully lifted off and transferred. Figure 12b shows the near field image, EL spectrum, and normalized output power of fabricated 8-μm micro-LEDs. Wu et al. demonstrated submicron dimension LEDs grown on Si without plasma damage. Through polarization and strain engineering, micro-LEDs showed ultra-stable, bright green emission with negligible QCSE^146^. Additionally, Chen, Jiang, and Zhang et al. fabricated pyramid-shaped micro LEDs employing the SAG technique^147–149^. Besides, Pandey, Liu, et al. have been developing SAE for high-performance nano-scale LEDs and have shown competitive performance^150–155^.

**Fig. 13:**
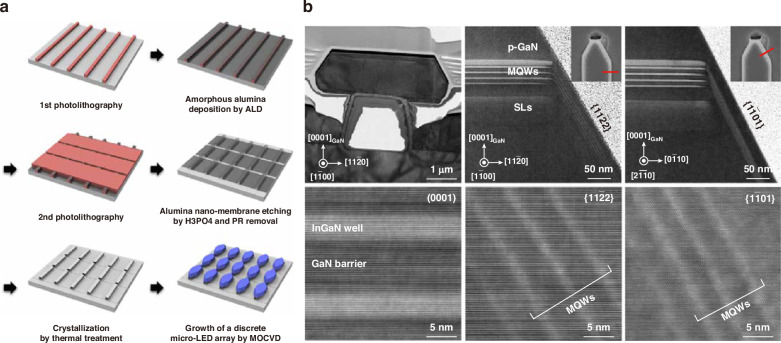
Core-shell micro-LEDs grown on sapphire nano-membranes. **a** Fabrication process flow, **b** TEM images of micro-LEDs grown on sapphire nano-membranes. Reproduced from ref. ^156^ © 2020, S. Lee et al. under the terms of the Creative Commons Attribution 4.0 License

Lee et al. proposed damage-free core-shell micro-LEDs grown on sapphire nano-membranes (NMs)^156^. The process is shown in Fig. 13a. First, a stripe-shaped photoresist was patterned on the sapphire substrate. Next, amorphous alumina was deposited by ALD and selectively etched by H_3_PO_4_. After all photoresists were removed, cavity-incorporated amorphous alumina was crystallized into a single crystalline sapphire by thermal annealing. Finally, the micro-LED array could be selectively formed on the sapphire NM without introducing an etching process. Although GaN was also grown on the spacing region between patterned Al_2_O_3_, the primary growth happened on the sapphire nano-membranes due to a more elevated position (~2 μm difference). Based on the above process, 4 μm × 16 μm micro-LEDs were demonstrated with suppressed threading dislocation density and higher IQE. Oh et al. further innovated the multiple-sapphire nanomembrane (MSNM) technique, which allowed micro-LED direct growth with the merits of plasma-free pixelation, stress weakening, reduced threading dislocation density, and rapid transfer^157^.

**Fig. 14:**
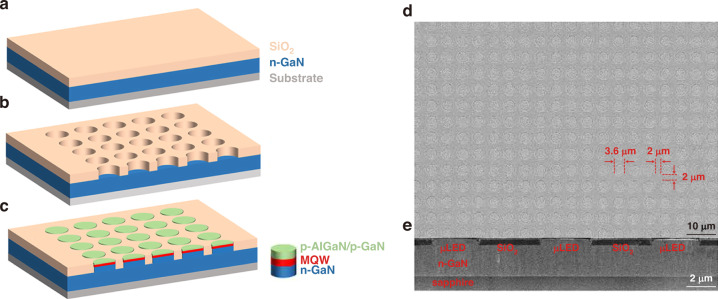
Confined selective epitaxy for ultra-small micro-LED direct growth. Schematic of the process flow (**a**) SiO_2_ deposition, (**b**) SiO_2_ mask patterning, and (**c**) micro-LED array overgrowth. **d** Top-view and **e** cross-sectional SEM images of micro-LED arrays. Reproduced from ref. ^158^ © 2020, American Chemical Society under the terms of the Creative Commons Attribution 4.0 License

Bai et al. proposed the confined selective epitaxy (CSE) approach for ultra-small micro-LED direct growth with precise dimension control^158^. For the conventional SAE method, thin dielectric masks were widely used to confine the epitaxy growth in the selective area until the growth was above the masks. The growth was then towards an unconfined manner along both vertical and lateral directions. However, for the CSE approach, epitaxial growth was always confined without the lateral direction of growth. Figure 14a–c shows the schematic of this innovation. A 500 nm PECVD-SiO_2_ was grown on the n-GaN template. With photolithography and etching processes, SiO_2_ microholes were formed. Subsequently, benefitting from the SiO_2_ patterning, the LED structure was selectively formed in the region without the SiO_2_ and insulated each other. The epitaxy growth thickness was around 500 nm, similar to that of the SiO_2_ mask. Therefore, the overall growth was confined entirely in the microholes, and the core-shell structure would not be formed under the CSE method. Without the lateral direction growth, the pixel size, shape, and spacing could be fully controlled as the initial design. Figure 14d, e show the top-view and cross-sectional SEM images of fabricated micro-LED arrays with a diameter of 3.6 μm and an interval of 2 μm. The peak EQE of 3.6 μm micro-green LEDs was around 6%. Feng et al. further utilized the CSE to achieve 2 μm InGaN-based red micro-LEDs with a wavelength of around 642 nm and peak EQE of 1.75%. Compared to the LED grown on the planar n-GaN template, the LED grown with the CSE method could achieve better strain relaxation and higher QW indium composition^159^. Besides, Esendag et al. demonstrated the leakage current of micro-LEDs could be significantly suppressed by employing the CSE process^160^. The above CSE method was further utilized to integrate micro-LEDs and HEMTs for display and communication applications^161^.

In summary, the selective area epitaxy offers new approaches for the future fabrication of efficient micro-LEDs and has significant research value. However, it should be noted that although the selective area epitaxy processes mentioned above do not involve any RIE damage, this does not mean that fully defect-free will necessarily be achieved at the interface. At pixel boundaries (such as the LED/SiO_2_ interface), the material quality and defect distribution may require further research and optimization.

### Ion implantation

Ion implantation is widely used in the semiconductor device process and has recently been employed for micro-LED pixelation by selectively insulating current injection regions. Xu et al. utilized a dual-energy F^−^ implantation to produce lattice disorder, selectively resulting in a resistive n-GaN electron injection layer. The 10 μm blue micro-LED array was fabricated, performing better than the non-treated broad-area LED^162^. Furthermore, after optimizing the implantation conditions, 6 μm blue micro-LEDs with more than 10% EQE were realized by Xu et al.^163^. Besides, Xu et al. demonstrated that heavy ions may be more suitable for micro-LED pixelation due to more stable optical and electrical isolation and better process reliability. The poor thermal stability of light hydrogen (H^+^) ions limited the device performance^164^. Furthermore, F^−^ ion implantation was also employed by Ye et al. to insulate the p-GaN for micro-LED pixelation^165^. Besides, Hsu employed Ar^+^ ion implantation to insulate the p-GaN for the micro-LED pixelation process^166^. Slawinska et al. used He^+^ implantation on the tunnel junction to define micro-LED aperture^167^. Noor Elahi et al. theoretically studied the current and light crosstalk in ion implantation fabricated micro-LEDs^168^.

**Fig. 15:**
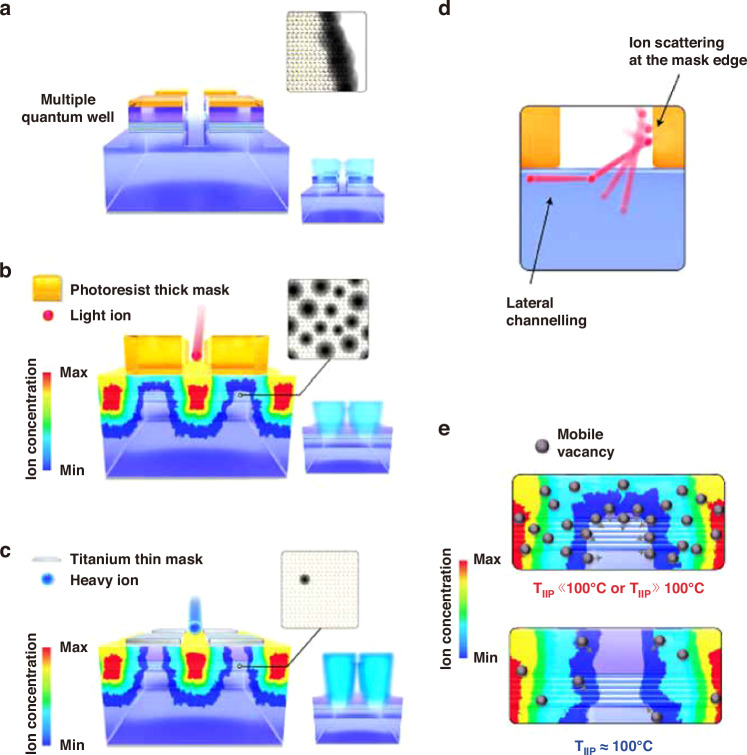
Tailored ion implantation technique for sub-micro LED fabrication. Schematic diagrams of (**a**) micro-LEDs fabricated by plasma etching, (**b**) micro-LEDs fabricated by ion-implantation, (**c**) micro-LEDs fabricated by tailored ion implantation, (**d**) ion scattering at the mask edge, and (**e**) effect of implantation temperature on the vacancy generation. Reproduced with permission from ref. ^169^ © 2021, J. Park et al

Park et al. reported a tailored ion implantation (TIIP) method to fabricate high-efficiency sub-micro LEDs with the line/space 0.5/0.5 μm, which corresponded to 8500 ppi resolution (RGB)^169^. Figure 15a–c shows the schematic illustrations of micro-LED pixelation by mesa-etching, IIP, and TIIP methods. The lateral spreading of implanted ions illustrated in Fig. 15d was significantly suppressed after optimizing ion implantation conditions (called TIIP). It was demonstrated that to achieve higher pixelation contrast, a thin implantation mask thickness was critical. A 0° tilt implantation angle and heavier ions were also significant. The implantation temperature should be well-optimized to suppress the vacancy generation, as shown in Fig. 15e. Besides, the minimal ion energy and implantation dose are advised to be used.

Moreover, Moon et al. developed a tailored He-based focused ion beam (FIB) irradiation technique to fabricate the micro-LED with a size down to 0.5 μm. The FIB irradiation resulted in optical quenching and electrical isolation effects for micro-LED pixel definition without suffering the conventional etching damage. It is a promising technique for maskless and etching-free ultra-small micro-LED fabrication^170^.

### Neutral beam etching and atomic layer etching

As discussed in the previous section, ion bombardment and UV photon irradiation are two significant factors contributing to sidewall damage in the conventional ICP-RIE process. However, these issues can be overcome by the neutral beam etching (NBE) technique. The NBE setup could effectively neutralize charged particles and block UV photon emission by a carbon aperture between the plasma and the etching chamber. Therefore, a neutral beam with precisely controlled kinetic energy is produced for micro-LED mesa etching^171–173^. Wang et al. employed the NBE to achieve 3.5 μm blue micro-LED with ultra-high peak EQE (37.5% measured by integration sphere and 26% measured by photodiode). Compared with the conventional ICP fabricated micro-LED, the NBE-fabricated device showed negligible surface nonradiative recombination. It eliminated the effect that micro-LED efficiency was seriously suppressed by the mesa size^174^. Lo and Hsu et al. also observed the size dependence of micro-LED efficiency is smaller in the NBE samples than in the ICP-RIE^175,176^. Based on the above results, the NBE process may become a significant technique in ultra-small micro-LED fabrication for tiny micro-displays. Furthermore, Ishihara, Ohori, et al. investigated the hydrogen iodide (HI) neutral beam as a new etching gas to replace the conventional Cl_2_. It was demonstrated that the HI-NB could provide a high etching rate without residual attachment on the etched surface. The defect generation was also significantly suppressed when using a HI-neutral beam for surface etching^177,178^. Therefore, it was expected to be a promising NBE source applied to micro-LED fabrication. However, Lo et al. found that NBE offers a more vertical mesa sidewall compared to ICP-RIE, which may reduce micro-LED LEE^175^. This issue needs to be noted in future optimizations of NBE-based micro-LED processes.

Atomic layer etching (ALE) could achieve material etching layer by layer via rapidly switching the plasma source and controlling etching power, which produces extremely low damage and a smooth surface after etching^179^. Therefore, it is considered a critical technique for potentially eliminating micro-LED sidewall effects^180^. We have recently verified that ALE can effectively remove the plasma damage layer in n-AlGaN and promote better metal contact^181^. However, there have been few reports about the ALE applied in micro-LEDs until now, and more exploration is needed.

### Metal-assisted chemical etching

Metal-assisted chemical etching (MacEtch) serves as an open-circuit anisotropic etching technique that avoids plasma damage. Its core mechanism involves three key steps: (1) photogenerated carriers, where the photons need to have a higher energy than the material’s bandgap; (2) the use of a metal catalyst to induce localized charge separation, leading to material oxidation; and (3) the removal of the oxidized material using an acid or base^182^. Recently, Chan et al. demonstrated the fabrication of micro-LEDs using the MacEtch process^182^. In their approach, HCl-based acid served as the etchant, and Ru was used as the metal catalyst. 5 μm blue micro-LEDs with an EQE exceeding 15% were successfully achieved. However, it was reported that p-type III-N materials were not efficiently etched using their MacEtch method. As a result, RIE was applied prior to MacEtch to remove the p-type materials.

### Selective insulation by plasma

**Fig. 16:**
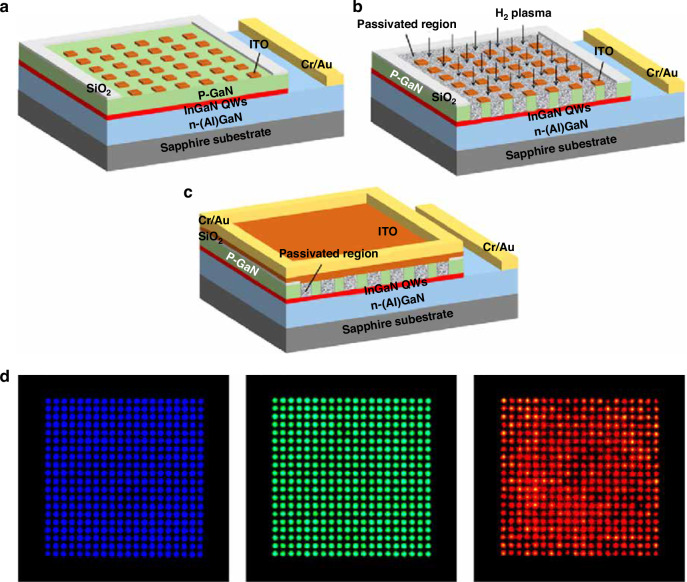
Selective passivation by H_2_ plasma for ultra-small micro-LED fabrication. Schematic of the process flow (**a**) ITO patterning as contact and treatment mask, (**b**) H_2_ plasma treatment, and (**c**) second deposition of ITO to connect pixels. **d** EL emission images of RGB monochromatic micro-LED arrays with a 5 μm size. Reproduced with permission from ref. ^184^ © 2021, Chinese Laser Press

In addition to the ion implantation method mentioned above, utilizing plasma treatment can also insulate specific areas of micro-LED, thereby achieving pixelation without physical insulation by etching. Massoubre et al. investigated the current blocking effect of CHF_3_, CF_4_, and H_2_ plasma treatment on p-GaN samples. The lowest p-GaN conduction was found after the CHF_3_ plasma treatment. The current blocking effect was explained from carrier concentration and metal contact perspectives: de-activation of Mg acceptors by hydrogen and increased metal-semiconductor barrier energy^183^. Zhuang et al. demonstrated ultra-small InGaN-based micro green, blue, and red LED arrays by selective passivation of p-GaN using PECVD-H_2_ plasma^184,185^. The pixel size was down to 4 and 5 μm. The schematics of fabrication processes are shown in Fig. 16a–c. In the process, the patterned ITO was used to block H_2_ plasma entering into pixels. Meanwhile, other areas of p-GaN were passivated by the H_2_ plasma and became insulated, thus suppressing hole injection from these regions. A second deposited ITO was then used to connect each pixel and spread the current. Figure 16d shows EL emission images of InGaN-based red, green, and blue micro-LED arrays with a pixel size of 5 μm. However, calculated from a modified TLM model, hydrogen atoms had a 1.76 µm diffusion length in this study, which theoretically limited the minimum pixel size. A significant degradation was found in micro-LEDs when the pixel size was reduced to 2.7 µm.

### Selective thermal oxidation

**Fig. 17:**
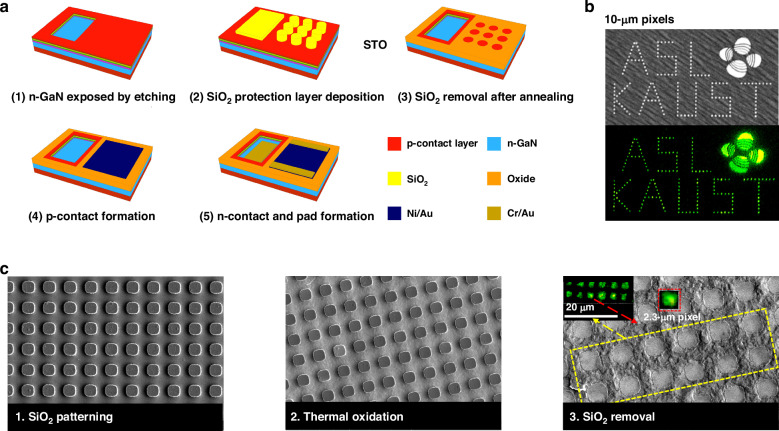
Selective thermal oxidation for micro-LED fabrication. **a** Schematic of the fabrication process flow, **b** 10 µm pixel display schematic diagram, **c** SEM images of 2.3 µm micro-LED pixelation by STO. Reproduced from ref. ^186^ © 2024, Z. Liu et al. under the terms of the Creative Commons Attribution 4.0 License

Recently, a novel etching-free technique, called selective thermal oxidation (STO), was proposed for the definition of micro-LED pixels^186^. The whole fabrication process is shown in Fig. 17a. The STO intentionally oxidized and thus inactivated areas between pixels, generating arrays of pixels without the use of plasma etching. Meanwhile, a thick SiO_2_ served as the protective layer to prevent the pixel areas from oxidation. Based on the STO process, the 10 µm pixel array exhibited a low leakage current density of 1.2 × 10^−6^ A cm^−2^ at −10 V voltage and a peak on-wafer external quantum efficiency of approximately 6.48%. The luminescent image of a pattern combined with 10 µm pixels is shown in Fig. 17b. Aiming at tiny micro-displays such as AR/VR, 2.3 µm micro-LED pixelation was preliminarily demonstrated, as shown in Fig. 17c. Besides, the STO can be directly applied to the fabrication of individual micro-LEDs and is fully compatible with the mass transfer process for full-color display. However, in selective thermal oxidation, the oxidation process may lead to the degradation of p-contact performance and device efficiency, even with SiO_2_ protection. Relevant solutions are expected to be proposed in the future.

In summary, various pixel definition methods without conventional plasma etching have recently been proposed. These methods offer more strategies for micro-LED fabrication. Due to the removal of the ICP-RIE process, micro-LED pixels exhibited unique performance and physical characteristics, which suggested that they are promising techniques for fabricating highly efficient micro-LEDs in the future. However, it is worth noting that these methods are still in the early stages of validation and development, and various issues remain as discussed above. More in-depth understanding of mechanisms and condition optimization of these methods are expected in the future to enhance device performance and solve limitations. Even with the introduction of “damage-free” concepts and techniques, we still need to continue developing “sidewall engineering” based on the RIE process. Advancing micro-LED fabrication technologies with and without RIE in parallel is beneficial for mutual comparison and competition.

## Conclusion

**Fig. 18:**
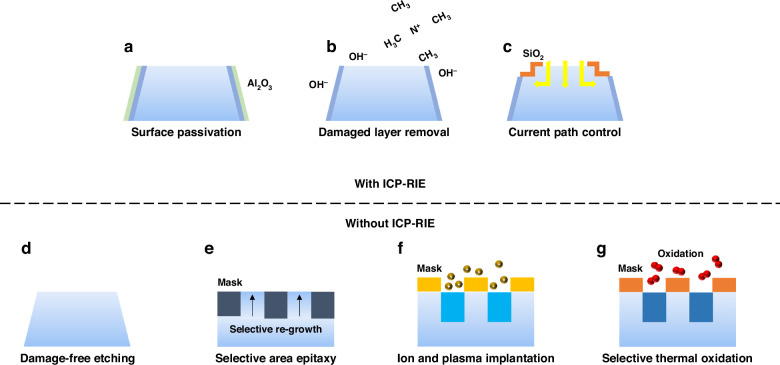
Advanced fabrication technologies for InGaN micro-LEDs. **a** Surface passivation, **b** damaged layer removal, **c** current path control, **d** damage-free etching, **e** selective area epitaxy, **f** ion and plasma implantation, **g** selective thermal oxidation

Figure 18a–g and Table 1 conclude advanced fabrication technologies for InGaN micro-LEDs discussed in this work. The cited references are classic examples of the techniques employed. More comprehensive references are provided in the previous discussion. Table 2 summarizes state-of-the-art blue micro-LED (1–10 μm) performance based on RIE mesa insulation and sidewall treatment. The symbol “~“ indicates that the data was re-calculated or estimated from the figures in the references and is provided for reference only. The information shows that optimizing etching conditions, removing sidewall damage layers, passivating the sidewall surface, etc. processes are crucial for achieving high-performance micro-LEDs. It is worth mentioning that Yu et al. achieved an EQE of 13.02% for 1 µm blue micro-LEDs, while Xu et al. achieved an EQE of 22.3% for blue micro-LEDs with a size of 1.3 × 1.5 µm^2^ ^56,129^. In addition, using similar process methods, Xu et al. and Yu et al. demonstrated EQE of 19.3% for 1.3 × 1.5 µm^2^ micro green LEDs and 9.57% for 1 µm micro green LEDs, respectively. For InGaN red micro-LEDs, poor material quality is the primary factor limiting device efficiency. Recently, Li et al. achieved a 5.5% EQE for a 10 µm micro-LED with a wavelength of 601 nm^86^. Li et al. also achieved a 4.5% EQE for a 5 µm InGaN red micro-LED^187^. Lim et al. demonstrated a 0.44% EQE for a 5 µm micro-LED with a wavelength of 643 nm^188^. Yu et al. achieved a 0.86% EQE for a 1 µm micro-LED with a wavelength of 613.6 nm on a GaN substrate^189^. Table 3 summarizes state-of-the-art micro-LED (1–10 μm) performance based on the RIE-free micro-LED fabrication approaches. It is worth highlighting that Xu et al. achieved an EQE of 20.4% for 10 μm blue micro-LEDs using an ion implantation approach^163^. Wang et al. realized an EQE of 37.5% for 3.5 μm blue micro-LEDs by utilizing a neutral beam etching technique^174^. These high-level validations fully demonstrate the effectiveness of the RIE-free micro-LED fabrication process. It is important to note that EQE is affected by several factors such as the epitaxial quality of the material and device packaging. As a result, the merits of the technique should not be evaluated solely based on EQE values. Both RIE-based sidewall engineering and RIE-free techniques offer valuable insights for the future development of high-performance micro-LEDs. These techniques hold potential for further advancement and may significantly impact the next-generation micro-LED display market.

**Table 1:** Advanced fabrication technologies for InGaN micro-LEDs

Techniques following ICP-RIE process	Remove damaged layer	KOH^28^	TMAH^32^	H_3_PO_4_^39^
		(NH_4_)_2_S^92^	NH_4_OH^94^	HF^100^
		HCl^101^	Octadecylthiol^104^	Parylene^105^
	Passivate sidewall surface	PECVD	SiO_2_^109^	
		ALD	Al_2_O_3_^115^	
			SiO_2_^34^	
			AlN^119^	
		Multi-layers	ALD-Al_2_O_3_/PECVD-SiO_2_^121^	
			Al_2_O_3_/ZnO^122^	
			Thermal ALD-HfO_2_ or SiO_2_/plasma-enhanced ALD-SiO_2_/thermal ALD Al_2_O_3_^123^	
		Sol-gel	SiO_2_^124^	
		Plasma or ion	N_2_ plasma^127^	
			N ion^128^	
			N_2_O plasma^129^	
		Thermal annealing	^131^	
	Control current path	Lateral confinement	SiO_2_ refill^135^	
			Ta_2_O_5_ refill^136^	
			Buried tunnel junction^137^	
			ITO/p-GaN junction^138^	
		Hole injection capability optimization^132^		
		Contact geometry^133^		
		p-GaN thinning^134^		
		H_2_ plasma^139^		
		Sidewall electrode^140^		
		Carrier localization^141^		
Techniques without ICP-RIE process	Direct epitaxy	Selective area epitaxy or growth^145^		
		Sapphire nano-membranes^156^		
		Confined selective epitaxy^158^		
	Ion implantation	F^−^ implantation^162^		
		Ar^+^ implantation^166^		
		He^+^ implantation^167^		
		He-based focused ion beam irradiation^170^		
	Neutral beam etching	Cl_2_ source^174^		
		Hydrogen iodide source^177^		
	Atomic layer etching	^180^		
	Metal-assisted chemical etching	HCl-based etchant and Ru metal catalyst^182^		
	Plasma insulation	CHF_3_ plasma treatment^183^		
		H_2_ plasma treatment^184^		
	Selective thermal oxidation	Air atmosphere^186^

**Table 2:** State-of-the-art blue micro-LED (1–10 μm) performance based on RIE mesa insulation and sidewall treatment

InGaN blue micro-LEDs	**Reference**	**Wavelength**	**Pixel size**	**EQE peak**
	^190^	447 nm	10 μm	40.2%
	**Operation voltage**	**Reverse leakage**	**EQE droop**	
	3.5 V at ~50 A cm^−2^	/	45.7% from *J*_EQE-peak_ (26 A cm^−2^) to 900 A cm^−2^	
	**ICP-RIE condition or description**			
	Top-down RIE from the ITO to the n-GaN.			
	**Techniques to mitigate the sidewall effect**			
	Omnidirectional reflector (ODR) via ion beam deposition, with alternating layers of SiO_2_ and tantalum Ta_2_O_5_ with a final layer of Al_2_O_3_.			
	**Reference**	**Wavelength**	**Pixel size**	**EQE peak**
	^10^	455 to 470 nm	2 μm	~13.4%
	**Operation voltage**	**Reverse leakage**	**EQE droop**	
	~2.7 V at 10 A cm^−2^	< 600 pA (detection limit)	~56.6% from *J*_EQE-peak_ (12 A cm^−2^) to ~1000 A cm^−2^	
	**ICP-RIE condition or description**			
	CH_4_/H_2_/Ar RIE removed ITO, and Cl_2_/N_2_ ICP etching removed InGaN/GaN layers. Subsequently, a short SiCl_4_ RIE treatment was used to improve ohmic contact.			
	**Techniques to mitigate the sidewall effect**			
	A room-temperature KOH treatment was conducted for around 30 min to eliminate plasma damage. Then, 20 nm of ALD-Al_2_O_3_ (trimethylaluminum, TMA + H_2_O) was then deposited at 300 °C to passivate the sidewall surface, followed by 250 nm PECVD-Si_3_N_4_ at 250 °C for electrical insulation.			
	**The performance of pixels with other sizes in this work**			
	For 5 and 10 μm blue micro-LEDs: EQE ~ 10.6% and ~9.4%			
	**Reference**	**Wavelength**	**Pixel size**	**EQE peak**
	^129^	466.6 nm	1.3 × 1.5 µm^2^	22.3%
	**Operation voltage**	**Reverse leakage**	**EQE droop**	
	~2.6 V at 10 A cm^−2^	~1 × 10^−7^ A cm^−2^ at −3 V	/ (EQE peak at ~12 A cm^−2^)	
	**ICP-RIE condition or description**			
	A low radio frequency power of 30 W has been opted to reduce plasma-induced sidewall damage.			
	**Techniques to mitigate the sidewall effect**			
	KOH and H_3_PO_4_ etching to reduce surface defects. 5 min N_2_O plasma treatment with the radio frequency power of 5 W to minimize defect density. 1 µm SiO_2_ layer as a passivation and planarization layer.			
	**Other characterizations of interest**			
	Luminance >1 × 10^7^ nits, light power densities > 4 × 10^4^ mW cm^−2^ at ~150 A cm^−2^			
	**Reference**	**Wavelength**	**Pixel size**	**EQE peak**
	^56^	~465 nm	1 μm	13.02%
	**Operation voltage**	**Reverse leakage**	**EQE droop**	
	~2.4 V at 10 A cm^−2^	/	~45.5% from *J*_EQE-peak_ (20 A cm^−2^) to 1000 A cm^−2^	
	**ICP-RIE condition or description**			
	ICP-RIE (Cl_2_/Ar/BCl_3_^191^) was used to etch GaN-based materials.			
	**Techniques to mitigate the sidewall effect**			
	200 nm-thick PECVD-SiO_2_ served as a passivation layer.			
	**The performance of pixels with other sizes in this work**			
	For 2 and 5 μm blue micro-LEDs: EQE ~ 15.6% and ~19.1%

**Table 3:** State-of-the-art micro-LED (1–10 μm) performance based on the RIE-free micro-LED fabrication approaches

Direct epitaxy	**Method**	**Wavelength**	**Pixel size, PPI**
	Confined selective epitaxy^158^	520 nm at 30 A cm^−2^	3.6 μm, 4536
	**Operation voltage**	**Reverse leakage**	**EQE peak**
	3.4 V at 60 A cm^−2^	1.3 × 10^−2^ A cm^−2^ at −4 V	6.0%
	**Other characterizations of interest**		
	Luminance peak>10^7^ cd m^–2^, IQE of 28%		
	**Method**	**Wavelength**	**Pixel size, PPI**
	Confined selective epitaxy^159^	642 nm at 10 A cm^−2^	2 μm, 7257
	**Operation voltage**	**Reverse leakage**	**EQE peak**
	~3.1 V at 10 A cm^−2^	/	1.75%
	**Other characterizations of interest**		
	Luminance peak > 3.5 × 10^7^ cd m^–2^		
Ion implantation	**Method**	**Wavelength**	**Pixel size, PPI**
	F^−^ implantation on n-GaN^163^	460 nm	10 μm, 1270
	**Operation voltage**	**Reverse leakage**	**EQE peak**
	2.83 V at ~16 A cm^−2^	~3.1 × 10^−6^ A cm^−2^ at −5 V	20.4%
	**Other characterizations of interest**		
	Output power density of 82.1 W cm^−2^ at 220 A cm^−2^ 31.7% EQE droops from *J*_EQE-peak_ (~40.8 A cm^−2^) to 220 A cm^−2^		
	**The performance of pixels with other sizes in this work**		
	For 6 µm pixels: 10.1% EQE peak, 8.4% EQE droops from *J*_EQE-peak_ (~113.3 A cm^−2^) to 220 A cm^−2^		
	For 8 µm pixels: 15.3% EQE peak, 17.9% EQE droops from *J*_EQE-peak_ (~63.7 A cm^−2^) to 220 A cm^−2^		
Neutral beam etching	**Method**	**Wavelength**	**Pixel size, PPI**
	Cl_2_-based NBE process^174^	451 nm	3.5 μm, /
	**Operation voltage**	**Reverse leakage**	**EQE peak**
	~3.0 V at 10 A cm−^2^	~1.2×10^−4^ A cm^−2^ at −4 V	37.5%
	**Other characterizations of interest**		
	26% efficiency droop from *J*_EQE-peak_ (3 A cm^−2^) to 0.01 A cm^−2^		
	**The performance of pixels with other sizes in this work**		
	For 6.5, 10.5, 20.5 μm pixels: EQE > 30%		
	For 6.5, 10.5, 20.5 μm pixels: 34.7%, 36.7%, 37.2% efficiency droop from *J*_EQE-peak_ (3 A cm^−2^) to 0.01 A cm^−2^		
Metal-assisted chemical etching	**Method**	**Wavelength**	**Pixel size, PPI**
	HCl-based acid chemistry^182^	445 nm	5 μm, /
	**Operation voltage**	**Reverse leakage**	**EQE peak**
	~9.8 V at 0.1 A cm^−2^	1.0×10^−4^ A cm^−2^ at −2 V	> 15.0%
Plasma insulation	**Method**	**Wavelength**	**Pixel size, PPI**
	H_2_ plasma treatment^185^	/(Green)	5 μm, 2822
	**Operation voltage**	**Reverse leakage**	**EQE peak**
	~2.5 V at 10 A cm^−2^	/	9.6%
	**Method**	**Wavelength**	**Pixel size, PPI**
	H_2_ plasma treatment^184^	666 nm at 11.5 A cm^−2^	5 μm, 2822
	**Operation voltage**	**Reverse leakage**	**EQE peak**
	3.6 V at 12 A cm^−2^	~1.3 × 10^−5^ A cm^−2^ at −4 V	/
	**Other characterizations of interest**		
	936 mW cm^−2^ at 115 A cm^−2^ with 632 nm emission		
	**Method**	**Wavelength**	**Pixel size, PPI**
Selective thermal oxidation			
	Air oxidation^186^	551 nm at 28 A cm^−2^	10 μm, 1270
	**Operation voltage**	**Reverse leakage**	**EQE peak**
	2.9 V at 10 A cm^−2^	1.2 × 10^−6^ A cm^−2^ at −10 V	> 6.48%

In summary, we have investigated the impact of plasma damage on InGaN-based micro-LEDs and analyzed related mechanisms and models. Various device processes to improve micro-LED efficiency have been reviewed. Besides, novel “damage-free” processes for micro-LED fabrication have been investigated. We aim this work to provide the community with a more profound knowledge of physics and solutions to the micro-LED sidewall effect. This will be beneficial for achieving high-efficiency, ultra-small, and low-cost micro-LEDs in the near future. The related techniques can extend to other III-nitride electronics and optoelectronics to optimize device performance.
